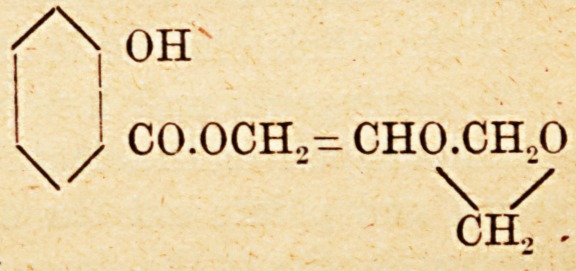# Remedies and Their Uses

**Published:** 1907-01-12

**Authors:** 


### REMEDIES AND THEIR USES.

### External Medication in Rheumatic Affections.

THE number of substances which have recently been introduced into medicine for outward application no doubt testifies to a very natural desire on the part of patients for some drug which can be immediately applied to the seat of pain. Not only does the idea that pain can be relieved by some topical application appear logical enough, and so commend itself *a priori* to the sufferer, but there is a subtle psychic relief in direct local action which will be appreciated by all who have undergone, from whatever cause arising, the tortures of acute pain. Most of the modern applications are salicyl compounds, and their use is based on the view that they are of the nature of direct antagonists to the cause of the disease. Mesotan, which is one of the best known, is methoxymethyl ester of salicylic acid—

It is a clear fluid, practically without odour, hardly soluble in water, but easily soluble in oil and ordinary organic solvents. It is decomposed in the presence of water, and thus must be kept carefully stoppered. There is a considerable amount of evidence that mesotan is a valuable local application in acute rheumatism; it is, at any rate, no inert body, as the main objection to its use is that it occasionally sets up somewhat severe dermatitis. In order to avoid this, Weil, in 1904, recommended the following method of application: The mesotan is never applied pure, but mixed with an equal part of olive oil; two or three teaspoonfuls are to be applied with a brush or *lightly* rubbed in every day, and no impervious material is to be superimposed. With these precautions no accident will occur, except in a few cases of special idiosyncrasy. Many reports since the publication of this paper have confirmed the writer's statements. Some have had no ill effects when mesotan, combined with two or four times its weight of vaseline, was rubbed into the skin. This method has, however, been known to set up violent dermatitis. Not only has mesotan been applied to the painful joints, tendons, ligaments, and muscles in acute and chronic rheumatism, but it has also been found useful in facial erysipelas, in pruritus, in hyperidrosis, and in the night-sweats of phthisis. It has also been painted round the eye in rheumatic iritis, but in this case aspirin was also given by the mouth.

*Esterdermasan, Rheumasan, and Rheumasol.*

The first two bodies are salicylic acid compounds on a soap basis, forming an ointment which contains about 10 per cent. of the active ingredient. The last is a brown fluid consisting of 10 per cent. salicylic acid, 10 per cent. petrosulphol, on a petroleum basis. They have been successfully employed in cases of acute rheumatism, and also in the so-called "muscular rheumatism," lumbago, and obstinate sciatica. The skin may first be washed with alcohol and ether to increase its absorptive power. Behr (*Therap. Monatsh.*, 1904) strongly advises massage with rheumasan over painful parts, and has not observed any symptoms of irritation of the skin or renal disturbance after prolonged use. The daily inunction may reach 75 to 150 grains. Besides the various rheumatic affections, it may be used in phthisis, neuralgia, migraine, and dry pleurisy. Rheumasol is applied in a similar manner, or may be painted on with a brush; it is employed for similar affections. Esterdermasan has also been used in gynæcological conditions by Wolff (*Klin. Therap. Wochenschr.*, 1906) as an analgesic. In chronic and acute parametritis, perimetritis, oöphoritis, etc., the abdominal walls were rubbed with the substance, and gelatin capsules containing 75 grains were placed in the vagina.

*Fetrosal.*

This substance, also known as velosan, is an ointment containing salicylic acid and salol in a special basis. Twenty-five per cent. of the salicylic acid may be recovered from the urine. The drug is said to be easily absorbed and non-irritant. It keeps well and has a pleasing odour; it has been used for rheumatic affections, and also in dermatology.

*Protosal.*

This is a colourless oily fluid, and is a compound of salicylic acid with glycerin-formal—

It is insoluble in glycerin itself, but can easily be dissolved in alcohol, fatty oils, or other organic solvents. Friedländer (*Therap. Monatsheft*, 1905) advises the following formula for application in rheumatic conditions:—

| | |
|---|---|
| Protosalis ... ... ... ... | 50 parts |
| Spiritus vini. rect. ... ... ... | 5 parts |
| Ol. olivæ ad ... ... ... ... | 100 parts |

The above preparations can be obtained from Merck, of Darmstadt.

### Pyramidon.

A proprietary title for dimethyl-amido antipyrin prepared by Meister Lucius and Bruning is a useful antipyretic which has been administered with very striking results in several typhoid epidemics in Germany, where it is believed to combine with antipyretic properties a considerable power of intestinal disinfection. Whether this is actually the case or not, the patients treated with pyramidon found the persistent headache in the early stages of typhoid very greatly relieved, and in many instances where insomnia was present the drug acted as a reliable hypnotic, while the temperature remained consistently lower than usual, even in the severer cases. Occasionally a red pigmentation is imparted to the urine by this drug, but there seems to be no pathological significance in the occurrence. As regards dosage 4 or 5 grains may be ordered to be taken every four or six hours, either in the form of powders or in water; it may also be obtained in tablet form.

## Figures and Tables

**Figure f1:**
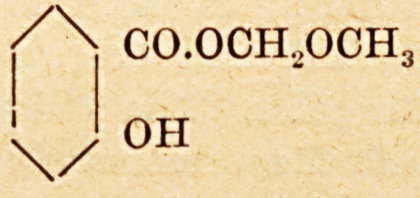


**Figure f2:**